# A Simplified Preoperative Assessment Predicts Complete Cytoreduction and Outcomes in Patients with Low-Grade Mucinous Adenocarcinoma of the Appendix

**DOI:** 10.1245/s10434-015-4446-y

**Published:** 2015-02-20

**Authors:** Sean P. Dineen, Richard E. Royal, Marybeth S. Hughes, Tara Sagebiel, Priya Bhosale, Michael Overman, Aurelio Matamoros, Paul F. Mansfield, Keith F. Fournier

**Affiliations:** 1 Department of Surgical Oncology, MD Anderson Cancer Center, Houston, TX USA; 2 Division of Gastrointestinal Oncology, National Cancer Institute, Bethesda, MD USA; 3 Division of Diagnostic Imaging, MD Anderson Cancer Center, Houston, TX USA; 4 Department of Gastrointestinal Medical Oncology, MD Anderson Cancer Center, Houston, TX USA

## Abstract

**Background:**

Complete cytoreduction with hyperthermic intraperitoneal chemotherapy (CRS/HIPEC) has been shown to improve survival in patients with low-grade mucinous adenocarcinoma (LGMA). However, incomplete cytoreduction exposes patients to significant morbidity without a similar survival benefit. Preoperative assessment of the ability to achieve CRS is therefore a critical step in selecting patients for CRS/HIPEC.

**Objective:**

The aim of this study was to develop and validate a preoperative scoring system to accurately predict the ability to achieve complete cytoreduction in patients with LGMA of the appendix.

**Methods:**

A simplified preoperative assessment for appendix tumor (SPAAT) score was developed based on computed tomography scan findings thought to predict incomplete cytoreduction. We applied the SPAAT score to patients with LGMA to determine the ability of the score to predict complete cytoreduction. This scoring system was then applied to a separate cohort of patients from a different institution. Sensitivity and specificity were determined for the SPAAT score. Survival was calculated and correlated with the SPAAT score and the completeness of cytoreduction score.

**Results:**

A SPAAT score of <3 is a significant predictor of complete cytoreduction in the derivation cohort. In the validation cohort, 40 of 42 patients with a SPAAT score <3 achieved a complete cytoreduction, for a positive predictive value of 95.2 % and a negative predictive value of 100 %. Additionally, the SPAAT score was a significant predictor of disease-free survival.

**Conclusions:**

The SPAAT score is a useful tool in the preoperative assessment of patients with LGMA who are under consideration for cytoreductive surgery. Prospective analysis of this scoring system is warranted to appropriately select patients who will benefit from CRS/HIPEC.

Appendiceal neoplasms are an uncommon entity, representing only 1 % of gastrointestinal malignancies. Adenocarcinomas represent about two-thirds of appendiceal malignancies, and spread of disease can result in accumulation of mucinous ascites, a clinical symptom known as pseudomyxoma peritonei.^1^,^2^ In such patients, survival can be significantly improved with appropriate treatment. Complete cytoreductive surgery (CRS) combined with hyperthermic intraperitoneal chemotherapy (HIPEC) can result in 5-year survival rates of 30–75 %.^3^–^6^ However, this survival benefit is highly dependent on obtaining a complete cytoreduction. The completeness of cytoreduction (CCR) can be characterized by the amount of remaining disease at the time of CRS/HIPEC. Patients with no visible disease (CCR0), or disease in which no remaining nodule is >2.5 mm (CCR1), have undergone a complete cytoreduction. Patients who have undergone an incomplete cytoreduction (CCR2 or 3) obtain significantly less benefit from the operation.^1^,^7^ However, the morbidity associated with CRS/HIPEC is not trivial, ranging from 28 to 49 %.^6^,^8^

Preoperatively identifying which patients are likely to undergo complete cytoreduction is important to reduce morbidity associated with incomplete surgery; however, preoperatively predicting these patients remains difficult. Imaging with computed tomography (CT) frequently understages the amount of disease present.^9^,^10^ Magnetic resonance imaging (MRI) may have some benefit, but the best outcomes still demonstrate an accuracy of 85 %.^11^ We present a novel scoring system, the simplified preoperative assessment for appendix tumor (SPAAT), which allows for a preoperative assessment of disease based on high-quality CT imaging, and predicts the ability to achieve a complete cytoreduction.

## Methods

All studies were conducted with the approval of the Institutional Review Board of the University of Texas MD Anderson Cancer Center (UTMDACC) and NCI Intramural Program.

### Derivation of Simplified Preoperative Assessment for Appendix Tumor (SPAAT) Score

**Fig. 1:**
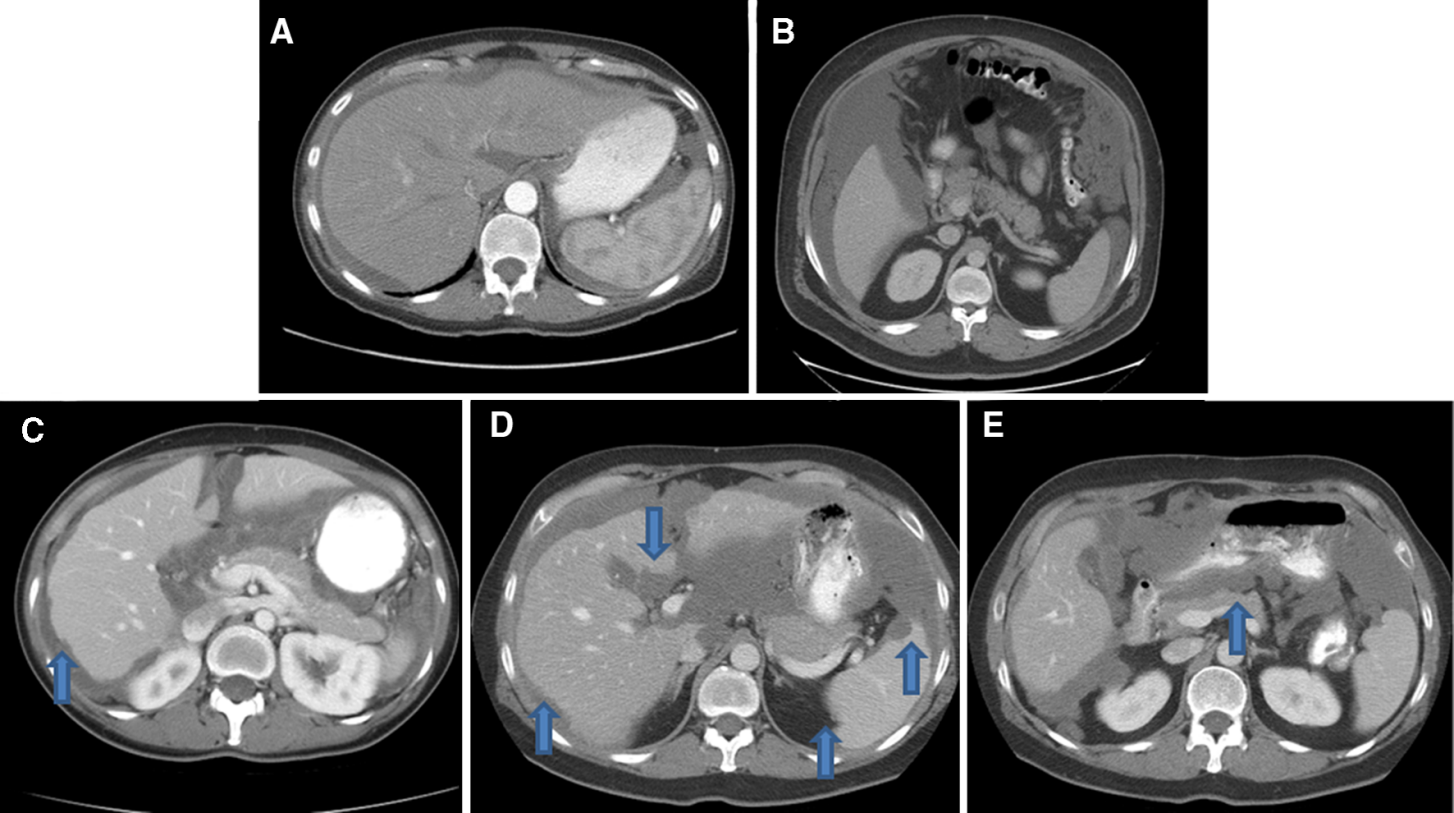
SPAAT scoring of visceral organs. **a** Mucinous ascites around the liver and spleen; however, the border is smooth without evidence of scalloping and this would be assigned zero points. **b** Mucinous ascites with a smooth spleen and pancreas border. Again, zero points are assigned. **c** CT scan findings of scalloping of the liver (*arrow*). **d** Liver and spleen scalloping (*arrows*). **e** Loss of the smooth pancreatic border with indentation of the organ (*arrow*), representing a CT finding that would score one point. *SPAAT* simplified preoperative assessment for appendix tumor, *CT* computed tomography

**Fig. 2:**
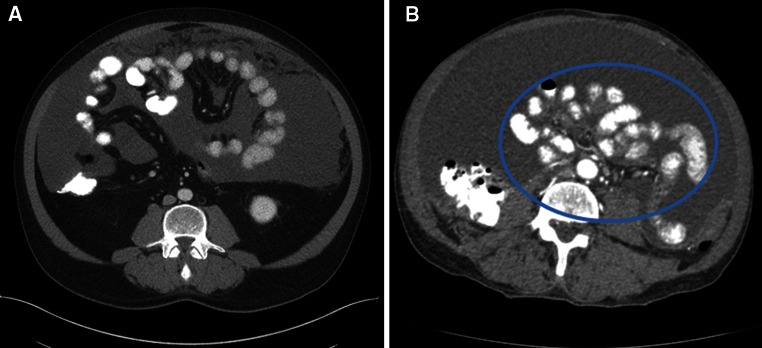
SPAAT scoring of the small bowel. *Panel* **a** demonstrates a patient with a significant amount of mucinous ascites, but the small bowel still appears to be floating freely. This would be assigned a zero for the SPAAT score. However, in *panel* **b** the patient demonstrates tethering of the small bowel, and would be given 3 points in the SPAAT system. *SPAAT* simplified preoperative assessment for appendix tumor

We developed a set of imaging criteria to predict complete cytoreduction based on preoperative CT Scans in patients with low-grade mucinous adenocarcinoma (LGMA) of the appendix treated at our institution (MD Anderson Cancer Center, Houston, TX, USA). Five anatomic locations were assessed and graded to reflect the volume and nature of the disease. The presence of scalloping was considered to be the important imaging feature; this feature is identified by the indentation of the organ by mucinous ascites. One point each was given to the presence of scalloping on the liver, spleen, pancreas, or portal vein (Fig. 1). Additionally, 0 or 3 points were assigned to the absence or presence of mesenteric foreshortening of the small bowel. In the presence of mucinous ascites, the small bowel appears to float to the abdominal wall in the absence of mesenteric foreshortening (Fig. 2a). Once the small bowel mesentery becomes involved with tumor, the mesentery foreshortens, causing the small bowel to appear tethered and cocoon-like on CT (Fig. 2b), a term known as ‘cauliflowering’ of the small bowel. This score was assigned as either a 0 or 3. The SPAAT scoring system allowed for scores ranging from 0 to 7. All CT scans were obtained with intravenous contrast.

### Derivation Cohort [MD Anderson Cancer Center (MDACC)]

The CT scans of 30 consecutive patients were evaluated between June 2008 and June 2009. The SPAAT score was calculated in a fashion blinded to the outcomes of the case, and analysis was subsequently performed to determine sensitivity and specificity.

### Validation Cohort [National Cancer Institute (NCI)]

Patients with LGMA treated at the National Cancer Institute (NCI) from 1997 to 2005 were identified. Patients with other histologic diagnoses were not included, and all patients were treated with the intention of complete cytoreduction and HIPEC. Radiologic and clinical data were retrospectively reviewed to identify the CCR, the SPAAT score, complications, and survival data. SPAAT scores were obtained by consensus review of two surgeons and two radiologists familiar with the treatment of patients with peritoneal disease, who were blinded to the outcome of the operation.

### Cytoreduction and Hyperthermic Intraperitoneal Chemotherapy

Patients at both institutions were treated with complete CRS involving resection of the entirety of gross disease and associated visceral resections, as needed.^12^ Intraperitoneal chemotherapy was administered using a closed technique at temperatures in excess of 40 °C. The choice of chemotherapy was either mitomycin C or cisplatin, and was determined by practices at each institution. A CCR score was determined by review of operative and clinical notes. If not indicated, the score was determined by review of the details of the operative note.

### Statistical Analysis

A true positive was defined as a SPAAT score which predicted a complete resection. For example, a SPAAT score <3 predicting a CCR0/1 resection would be considered a true positive, and a SPAAT score <3 in a patient with a CCR2/3 resection would be considered a false positive. A SPAAT score ≥3 predicting a CCR2/3 resection would be a true negative. In this manner, sensitivity, specificity, positive predictive value (PPV), and negative predictive value (NPV) were determined for SPAAT scores using cutoff values of 2, 3, or 4. Sensitivity was defined as the number of patients with optimal cytoreduction and a SPAAT score <3 (true positives, *n* = 40) divided by the total number of patients who had optimal cytoreduction (condition positive, *n* = 40). Specificity was defined as the number of patients with an incomplete cytoreduction with a SPAAT score ≥3 (true negatives, *n* = 28) divided by the total number of patients with incomplete cytoreduction (condition negative, *n* = 30). PPV and NPV were likewise determined by standard methods.

Overall survival (OS) data were calculated using the date of surgery to the date of last follow-up or death. Disease-free survival (DFS) was determined using the date of surgery and the date of first recurrence, while survival was compared using Kaplan–Meier methods. Prognostic factors were examined using univariate and multivariable Cox regression, and categorical variables were compared using the *χ* ^2^ analysis. Receiver operating curves were created to estimate the area under the curve. Data were collected in a database and analyzed using SPSS version 21 software (IBM Corporation, Armonk, NY, USA).

## Results

### Derivation Cohort (MDACC)

A SPAAT score was determined for 30 patients with low-grade appendiceal (LGMA) cancers treated with attempted CRS/HIPEC at the MDACC from June 2008 to June 2009. Overall, 27 patients had sufficient data available for analysis, and SPAAT scores ranged from 0 to 6. We determined that using a SPAAT score cutoff of 3 identified 23 patients with complete resection and 4 patients with incomplete resection, for a sensitivity and specificity of 100 %. The various sensitivity and specificity of cutoff values 2, 3, or 4 are shown in Table 1.

**Table 1:** SPAAT score of 3 demonstrates best results in derivation and validation cohorts

Cutoff	Sensitivity (%)	Specificity (%)	PPV (%)	NPV (%)	Spearman
Derivation cohort (*n* = 27)					
SPAAT ≥ 2	100	36.4	69.6	100	0.503
SPAAT ≥ 3	100	100	100	100	1.0
SPAAT ≥ 4	92	100	100	50	0.678
Validation cohort (*n* = 70)					
SPAAT ≥ 2	62.5	93.3	92.6	65.1	0.568
SPAAT ≥ 3	100	93.3	95.2	100	0.943
SPAAT ≥ 4	100	70	81.6	100	0.756

*NPV* negative predictive value, *PPV* positive predictive value, *SPAAT* simplified preoperative assessment for appendix tumor

### Validation Cohort (NCI)

In an effort to validate the scoring system on a larger group of patients, external to our institution, we then applied the scoring system to 70 patients treated at the surgery branch of the NCI. The median age of patients in this cohort was 50 years. There were no differences in demographics with respect to age, sex, ethnicity, or performance status between patients with SPAAT scores <3 versus patients with SPAAT scores ≥3. Overall, patients had a good performance status, with 97 % of patients demonstrating an eastern cooperative oncology group (ECOG) status of 0. Median follow-up was 52.36 months.

**Fig. 3:**
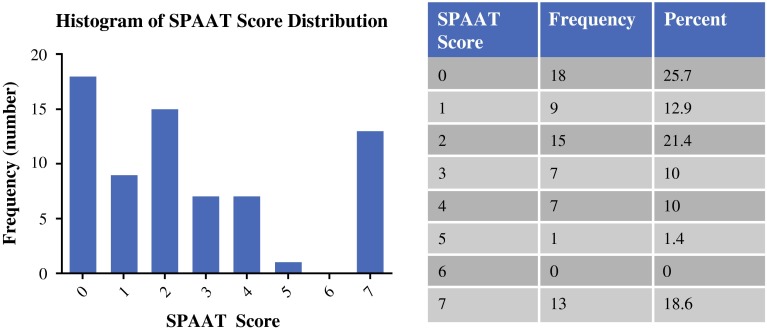
Distribution of SPAAT scores in the validation cohort (*n* = 70). Scores in the validation cohort ranged from 0 to 7. Overall, 42 patients (60 %) demonstrated an SPAAT score <3, and 28 (40 %) had scores ≥3. *SPAAT* simplified preoperative assessment for appendix tumor

Forty-two patients had a SPAAT score <3, and 28 had a SPAAT score ≥3. SPAAT scores ranged from 0 to 7; the distribution of scores is shown in Fig. 3. Data on sensitivity, specificity, PPV, NPV, and accuracy for SPAAT values 2, 3, and 4 are shown in Table 1. Using a cutoff of 3, the SPAAT score showed the greatest sensitivity and specificity; therefore, for further analysis, the cutoff of a SPAAT score <3 was used. Next, a receiver operating characteristic curve was generated for the SPAAT score, with an area under the curve of 0.947 (95 % confidence interval 0.874–1.00; *p* < 0.001).

**Fig. 4:**
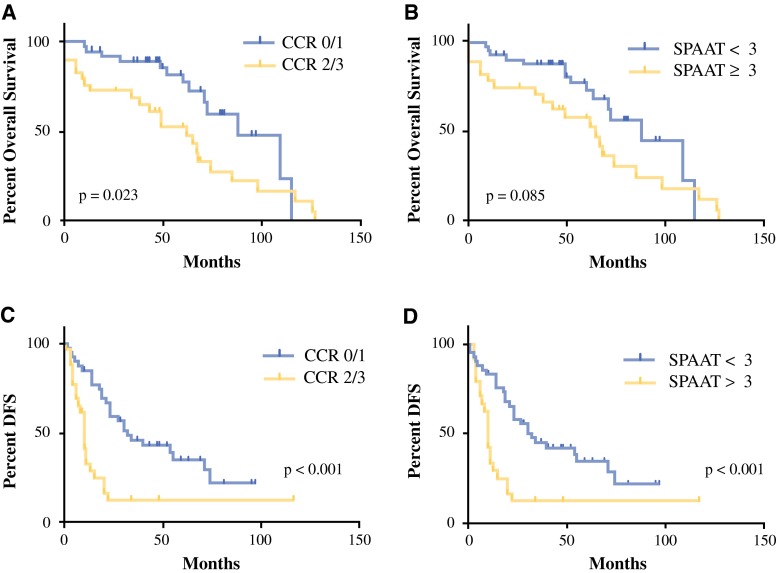
Complete cytoreduction is associated with improved OS and DFS. **a** OS is significantly higher in patients with complete cytoreduction compared with those with incomplete cytoreduction. **b** Patients with SPAAT scores <3 demonstrated improved OS compared with those with an SPAAT score ≥3; however, this did not reach statistical significance. **c** DFS was compared in the validation cohort between patients with a complete cytoreduction and those without. The median DFS was significantly longer in patients with CCR0/1 resection (*p* < 0.001). **d** DFS for patients based on SPAAT score. The survival curves for patients with CCR1/2 resection are nearly identical to those with an SPAAT score <3. *CCR* completeness of cytoreduction, *DFS* disease-free survival, *OS* overall survival, *SPAAT* simplified preoperative assessment for appendix tumor

For survival analysis, we first analyzed the cohort for the effect of complete CRS (CCR0 or 1). As depicted in Fig. 4, we showed that patients with a complete cytoreduction have improved OS and DFS compared with those with incomplete cytoreduction. Median OS was 88 ± 14.22 months in the CCR0/1 group versus 62 ± 11.96 months in the CCR2/3 cohort (*p* < 0.05). Median DFS was 32 ± 7.7 months in the CCR0/1 cohort compared with 10 ± 1 month in the incomplete cytoreduction group (*p* < 0.001). Subsequently, we then assessed our hypothesis that SPAAT scores would correlate with survival outcomes. Figure 4 demonstrates a significant difference in DFS, and a trend toward a difference in OS, between those patients with a SPAAT score <3 and those with a SPAAT score >3 (DFS: 32 ± 5.3 vs. 10 ± 1 months, *p* < 0.001; OS: 88 ± 14.4 vs. 65 ± 12.1 months). There were three 30-day mortalities (4.3 %), all in patients with a SPAAT score >3. Significant complications (Clavien 2, 3, 4) were not statistically different between groups, although were more common in the group with a SPAAT score ≥3 (34.6 % compared with 23 % in the group with a SPAAT score <3).

## Discussion

In this study, we demonstrated a simple preoperative scoring system that accurately predicts the ability to obtain complete cytoreduction in patients with LGMA. The scoring system is based on high-quality CT imaging and because of the association with complete cytoreduction, the SPAAT also correlates with survival outcomes. The importance of complete cytoreduction has been demonstrated in several studies.^1^,^4^,^13^ For example, Chua et al. reviewed over 2000 patients with appendiceal cancer from multiple institutions, and demonstrated a 24 % 5-year survival with incomplete cytoreduction compared with approximately 80 % for patients undergoing a CCR0/1 resection.^1^ Similarly, Baratti et al. showed a significant difference in both OS and DFS for patients undergoing incomplete cytoreduction.^13^ Our results showing a 5-year OS of 72.6 % in patients undergoing a complete cytoreduction are in line with these previous reports and demonstrate the importance of patient selection for surgery as incomplete cytoreduction has less benefit for patients. Our results for morbidity are also within published ranges. Thus, our model of predicting those patients most likely to achieve complete cytoreduction would appear to lead to the most clinical benefit, while minimizing morbidity.

It is important to note that our scoring system is a marker of overall disease severity rather than a predictor of where a patient will fail. There is a more direct correlation of small-bowel tethering and unresectable disease, thus the higher score for this finding. However, scalloping of the spleen leading to the assignment of a point on our scoring system does not suggest a patient is likely to fail in the left upper quadrant (as the spleen is easily resected in situations of significant disease). However, this is a preoperative marker of the extent, and possibly the aggressiveness, of disease one will encounter upon exploration. Likewise, our scoring system does not assign points for disease in the pelvis. This is not to suggest patients do not fail in the pelvis, but rather that we have not found imaging characteristics that predict the inability to achieve complete cytoreduction.

A scoring system somewhat similar to the SPAAT score has been developed for patients with advanced ovarian cancer. This system, designated the predictive index value (PIV) is based on laparoscopic evaluation of eight anatomic locations.^14^,^15^ A score ≥8 is an accurate predictor of the inability to achieve complete cytoreduction. We have developed our score using preoperative imaging with the intention of sparing the complications associated with laparoscopy. Our ability to predict those patients who will achieve complete cytoreduction is higher than that achieved with the PIV.^14^

We currently use the SPAAT score as an adjunct in the decision-making process for patients with LGMA. A SPAAT score ≥3 is not an absolute contraindication to operation; however, it is valuable to be able to set expectations prior to the operation. An incomplete cytoreduction may still benefit the patient with relief of symptoms, but the survival benefit is significantly less than for those patients with a complete cytoreduction.^1^,^16^ The SPAAT score allows for preoperative discussions to focus on the intent of operation, i.e., palliative versus intent for complete cytoreduction. Patients with significant comorbidities and a SPAAT score ≥3 would likely be counseled that the risks of operation outweigh the benefits. The converse is also true. Thus, a patient with mild to moderate comorbid conditions and with a SPAAT score <3 is likely to achieve complete cytoreduction and the survival benefit associated with such. Hence, an aggressive approach to operation may be warranted in this setting.

The limitations of our study include the retrospective nature of the data collection and the associated limitations of such studies. At the time of the study, we did not have consistent use of preoperative tumor markers to correlate the carbohydrate antigen (CA) 19-9, carcinogenic embryonic antigen, or CA-125 levels with imaging findings. Additionally, we only included patients with an excellent performance status (97 % ECOG 0). It is our practice, and that of others, to be highly selective with regard to offering CRS/HIPEC, but this limits the applicability of the scoring system to less fit patients. Most notably, the designation of a complete cytoreduction is based on leaving no visible disease >2.5 mm in size. This is somewhat subjective as not every site of disease is measured. As such, this may lead to bias as surgeons may have been more likely to designate a resection as CCR2 in the setting of more advanced disease on preoperative imaging. However, this was the rationale for the validation cohort. In this cohort, the CCR scores were derived independently of knowledge of the SPAAT score. We included only LGMA patients in this analysis. The SPAAT score should not be generalized to patients with high-grade appendiceal cancer at this time.

## Conclusions

Our data showed that the SPAAT score can reliably be obtained from high-quality preoperative CT scan. Using a cutoff of <3 points, we demonstrated an accuracy of 97.14 % in determining which patients would undergo a complete cytoreduction. As CCR is the predominant factor determining treatment success in patients with LGMA, we suggest that this scoring system can be useful in the preoperative decision-making process. Prospective trials utilizing the SPAAT score are warranted.
